# Can peripheral psychophysiological markers predict response to exposure-based cognitive behavioral therapy in youth with severely impairing irritability? A study protocol

**DOI:** 10.1186/s12888-023-05421-4

**Published:** 2023-12-11

**Authors:** Lana Ruvolo Grasser, Trinity Erjo, Matthew S. Goodwin, Reut Naim, Ramaris E. German, Jamell White, Lisa Cullins, Wan-Ling Tseng, Joel Stoddard, Melissa A. Brotman

**Affiliations:** 1 Neuroscience and Novel Therapeutics Unit, Emotion and Development Branch, National Institute of Mental Health, National Institutes of Health, Bethesda, MD USA; 2 Bouvé College of Health Sciences, Northeastern University, Boston, MA USA; 3 School of Psychological Sciences, Tel-Aviv University, Tel-Aviv, Israel; 4 Yale Child Study Center, Yale School of Medicine, New Haven, CT USA; 5 Department of Psychiatry and Biomedical Informatics, University of Colorado, School of Medicine, Aurora, CO USA

**Keywords:** Irritability, Cognitive behavioral therapy, Exposure, Heart rate, Heart rate variability, Electrodermal activity, Children and adolescents, Intervention

## Abstract

**Background:**

Irritability, an increased proneness to anger, is a primary reason youth present for psychiatric care. While initial evidence supports the efficacy of exposure-based cognitive behavioral therapy (CBT) for youth with clinically impairing irritability, treatment mechanisms remain unclear. Here, we propose to measure peripheral psychophysiological indicators of arousal—heart rate (HR)/electrodermal activity (EDA)—and regulation—heart rate variability (HRV)—during exposures to anger-inducing stimuli as potential predictors of treatment efficacy. The objective of this study is to evaluate whether in-situ biosensing data provides peripheral physiological indicators of in-session response to exposures.

**Methods:**

Blood volume pulse (BVP; from which HR and HRV canl be derived) and EDA will be collected ambulatorily using the Empatica EmbracePlus from 40 youth (all genders; ages 8-17) undergoing six in-person exposure treatment sessions, as part of a multiple-baseline trial of exposure-based CBT for clinically impairing irritability. Clinical ratings of irritability will be conducted at baseline, weekly throughout treatment, and at 3-month and 6-month follow-ups via the Clinical Global Impressions Scale (CGI) and the Affective Reactivity Index (ARI; clinician-, parent-, and child-report). Multilevel modeling will be used to assess within- and between-person changes in physiological arousal and regulation throughout exposure-based CBT and to determine whether individual differences are predictive of treatment response.

**Discussion:**

This study protocol leverages a wearable biosensor (Empatica) to continuously record HR/HRV (derived from BVP) and EDA during in-person exposure sessions for youth with clinically impairing irritability. Here, the goal is to identify changes in physiological arousal (EDA, HR) and regulation (HRV) over the course of treatment in tandem with changes in clinical symptoms.

**Trial registration:**

The participants in this study come from an overarching clinical trial (trial registration numbers: NCT02531893 first registered on 8/25/2015; last updated on 8/25/2023). The research project and all related materials were submitted and approved by the appropriate Institutional Review Board of the National Institute of Mental Health (NIMH).

## Background

Irritability is characterized by an increased proneness to anger, frustration, and temper outbursts [12, 53]. Irritability is one of the most common reasons youth present to psychiatric care and predicts anxiety and depression longitudinally ([12, 89, 90, 103]). Despite this, treatments for irritability are limited. One promising treatment approach, based on our neurobiologically informed understanding of irritability, is exposure to anger-inducing events [73]. Preliminary evidence demonstrates the efficacy of exposure to anger-inducing events (as part of exposure-based cognitive behavioral therapy, CBT) as a treatment for irritability [69]. However, findings based on clinical measures do not provide a full picture of the mechanisms through which exposure-based CBT may exert its effects. The specific physiological mechanisms of exposure therapy on irritability remain unknown. Peripheral psychophysiology may be one level of analysis through which to link therapeutic processes to clinical improvements [13]. As new treatments for clinically impairing irritability are prioritized, an understanding of predictors and mechanisms associated with improvement can help guide further development and refinement.

It is essential to understand the pathophysiological mechanisms of improvement in response to treatments for several reasons. First, a substantial portion of youth do not sufficiently benefit from evidence-based treatments like CBT [26]. Furthermore, there is a paucity of CBT studies that target anger in youth from which to derive an understanding of clinical efficacy [31, 48, 69, 73, 94]. Approximately 22% to 50% of youth undergoing CBT for anxiety disorders or posttraumatic stress—both of which include irritability as a diagnostic symptom—do not respond or do not reach remission [46, 51, 57, 79, 88]. In terms of anger, irritability, and related diagnoses, (e.g., attention-deficit/hyperactivity disorder, ADHD, and disruptive mood dysregulation disorder, DMDD), some medications and psychotherapeutic interventions have shown promise; however, clinically relevant impairment still seems to be maintained in a significant number of youth [5, 6, 21, 32, 93, 94, 103, 105]. Treatment non-response may be due to a lack of knowledge regarding the physiological mechanisms mediating treatment response [55]. Indeed, heterogeneity in not only symptom profiles but also underlying biological phenotypes (that may be causal or a result of different symptoms) may contribute to the observed variation in patient response to treatment and the need for different treatments [22, 59]. This is especially relevant in the case of irritability, as this transdiagnostic psychiatric phenotype cuts across 15 DSM-5 diagnoses [20]). Thus, there is a need to optimize approaches to enhance treatment efficacy [26].

Exposure therapy relies on extinction principles wherein emotionally evocative, salient stimuli are presented in a clinical context to evoke naturalistic verbal, behavioral, physiological, and emotional responses [52]. When the memory or representational structure is recalled, it becomes available for modification/updating [62]. As this ‘representational structure’ of the feared/anger-inducing component is repeatedly evoked, new information can be integrated, leading to the modulation and reduction of fear or anger [29]. Typically, this approach is examined using fear stimuli in the context of anxiety disorders. Given overlapping neural circuitry mediating anger and fear ([71, 72]), we anticipate that evoking stimulus-driven anger/frustration in the clinical setting will elicit physiological responses indicative of the corresponding negative affective representational structure. Through repeated exposure to anger-inducing stimuli, youth with clinically impairing irritability have the opportunity to practice (1) tolerance of negative affective states and (2) inhibitory control of their reactions to blocked goal attainment and negative affective states (e.g., not having an outburst [48]). Initial activation, as well as within- and between-session changes in physiological responses, can serve as indicators of learning and integration of competing inhibitory signals to modify and regulate the existing representational structure [1].

Peripheral psychophysiological signals—including heart rate (HR), heart rate variability (HRV), and electrodermal activity (EDA)—are measurable indicators of autonomic nervous system (ANS) functioning broadly implicated in psychiatric phenomena [61]. In youth, physiological arousal at rest is associated with severity of irritability; similarly, both subjective and physiological arousal in response to affective stimuli is associated with severity of irritability [15, 81]. Increased cardiovascular activity (HR) and decreased regulation (HRV) during inhibitory control are also associated with greater severity of irritability in youth, pointing to the potential for peripheral physiological biomarkers of irritability [70]. Objective psychophysiological signals like HR, HRV, and EDA have a place in the assessment of psychology across cognitive, social, and functional domains that, by nature, overcome issues contributing to biased self-reports and over/underreporting of subjective symptoms [37, 58].

HR, HRV, and EDA are linked to corticolimbic circuity (e.g., hypothalamus, amygdala, ventromedial prefrontal cortex (vmPFC) [17, 97]) involved in learning, emotion regulation, and pediatric psychopathology [26]. These structures are the primary brain targets of exposure-based CBT, and psychophysiological measures could be proxy indicators of the effectiveness of CBT in modifying these targets [97]. In the context of exposure-based therapies, physiological measures can be used as (1) predictors of treatment response [9, 60, 80, 86], (2) evidence for activation of emotions (e.g., fear, anger) during exposure [43, 44, 99], (3) an index of gradual habituation within sessions, and 4) quantifiable changes in arousal and regulation across sessions [11, 24, 78, 86]. Below, we briefly summarize the measurement of HR, HRV, and EDA in clinical settings to provide background and rationale for a study of changes in peripheral psychophysiology over the course of exposure-based CBT for youth with clinically impairing irritability.

### Electrodermal activity

Electrodermal activity (EDA) is an objective measure of autonomic and emotional arousal/reactivity [10, 17, 54] associated with sympathetic nervous system (SNS) activity [4]. Because sweat glands are innervated by sympathetic nerves alone, EDA is considered a purer metric of sympathetic activity [101]. Sweat gland activity increases with stress response. It serves as a measure of sympathetic activation [35] and can be detected as changes in conductance and electrical potentials using electrodes placed on the skin’s surface [87]. The amygdala directly mediates the expression of skin conductance responses to arousing (fear- and anxiety-provoking) stimuli [36].

EDA is altered in the context of psychopathology, and differences in EDA may indicate psychopathologic severity [36]. EDA has been reliably measured in infants, children and adolescents, and adults [42, 64, 65, 67, 75]. EDA is transdiagnostically implicated in youth psychopathology, e.g., disordered eating [50], posttraumatic stress [30], aggression [28], and conduct problems [23]. EDA has also been used to measure autonomic activation during laboratory tasks and in clinical settings as an indicator of emotional reactivity [7], extinction training [4], therapeutic response [19, 62, 100], and future psychopathology [40, 41].

### Heart rate

Heart Rate (HR) is another measure of ANS activity [17] that can be assessed in tandem with EDA to provide a more robust understanding of the physiological processes implicated in psychopathology and potentially underlying therapeutic change [70]. HR is defined as the number of beats per minute and increases with stress and emotional arousal [3, 104]. Increased tonic cardiovascular function and reactivity are repeatedly implicated across psychopathology, including panic disorder [25], conduct disorders [15], and posttraumatic stress disorder [11].

When measured in the lab, mean HR reliably maps onto normative emotional responses [92] and indicates the efficacy of exposures for triggering a physiological response in both adults and youth [44]. HR is also responsive to treatment—decreases in HR have been observed for adults with posttraumatic stress disorder (PTSD) undergoing prolonged exposure (PE) [60], as well as veterans with PTSD undergoing either virtual reality exposure therapy or PE [11].

### Heart rate variability

HRV can be used to measure a person’s potential to adapt to a challenge (baseline HRV) and the dynamic processes associated with responding in real time (HRV reactivity) [55]. HRV is defined as the variability between heartbeats over time and has been shown to decrease with stress. HRV is broadly considered to be a marker of psychological well-being and cardiovascular fitness and is a significant predictor of mortality [16]. Decreased HRV is associated with increased risk for psychiatric conditions (bipolar disorder, schizophrenia, ADHD, anxiety disorders, and depression) [16, 34, 86, 96]. More specifically, HRV has been previously linked to emotion regulation—an important skill to manage emotions, like anger, and modulate behavior. Previous work has shown that respiration rate induces corresponding heart rate oscillations that may prompt synchronized oscillatory activity in the brain and enhanced functional connectivity between brain regions associated with emotion regulation [63]. For example, across age groups, a higher root mean square of successive differences between R waves (RMSSD) has been associated with higher connectivity between the medial prefrontal cortex and amygdala [63]. Further, in both older and younger adults, HRV has been linked to functional blood oxygen level-dependent (BOLD) signal change in the the left orbitofrontal cortex and left anterior cingulate cortex, as well as cortical thickness of this region—both regions implicated in emotion regulation [107]. Like HR, HRV is responsive to treatment—evidence in adults suggests that HRV can serve as an indicator of therapeutic engagement [99] and clinical change [78, 99], whereby increases in HRV are associated with reduced risk profiles across samples [96]. This is likely because increased HRV enables greater autonomic flexibility, i.e., adaptability in the face of challenge.

Derived from the research on fear and related disorders [24], in the context of treating irritability, changes in emotion regulation may reflect changes in the information structure of anger-inducing stimuli, and HRV can serve as an index for this. Previous work has identified a negative association between HRV and irritability during a frustrating, rigged inhibitory control task [70]. Irritability, notably, is an expression of emotion dysregulation [74]. Thus, we hypothesize that increased HR and EDA and decreased HRV in youth with irritability will be associated with response to CBT [70].

### Summary and Hypotheses

Psychophysiology has played a role in distinguishing the phenotype of clinically impairing irritability, whereby youth with clinically impairing irritability report more arousal during an affective task [81]. As highlighted above, elevated arousal and decreased regulation at the level of the ANS are broadly implicated in psychopathology, including irritability. Probing variation in arousal and regulation as measured by HR, HRV, and EDA has the potential to elucidate mechanisms underlying exposure-based treatment based on principles of extinction, prediction of individual treatment response, and evaluation of treatment efficacy.

The aim of this proposed study is to assess in vivo psychophysiology—HRV, HR, and EDA—across in-person exposure-based CBT sessions for youth with clinically impairing irritability. The trial will include youth requiring treatment for a primary concern of severe and impairing irritability in multiple domains that cut across DSM-5 diagnostic categories. Our goal is to identify potential putative psychophysiological mechanisms of treatment efficacy. We consider three psychophysiological signals, as they are differentially regulated and represent variant psychological manifestations [62].

Our primary hypotheses are the following: (1) HRV will show an increase over time across sessions, reflecting improved regulation; (2) HR will show a reduction over time between sessions, reflecting decreased arousal; (3) EDA will show a reduction over time between sessions, also reflecting decreased arousal; and (4) these changes will each be associated with measurable improvement in symptoms over the course of treatment.

Post hoc exploratory analyses will probe causality, as indicated by temporal associations. That is, we will assess whether changes in psychophysiology precede and predict changes in symptoms over the course of treatment or vice versa using cross-lagged panel models. Of note, a consideration that affects most hypotheses is that blunted HR and EDA at initial exposure may be maladaptive [77, 83], possibly indicative of avoidance. Higher HR and EDA at the beginning of treatment may demonstrate both sufficient engagement in exposures and sufficiency of exposures to evoke a level of physiological arousal equivalent to that experienced in a naturalistic setting. Additionally, increased HR/EDA and decreased HRV at initial in vivo exposure may reflect a greater ability of youth to engage in context-appropriate responses and, thus, more likely to have better treatment outcomes. At the same time, higher HRV could reflect active avoidance [9]. Accordingly, we will adjust our models using formal comparison by information criteria to account for potential nonlinear changes caused by initial avoidance.

Aberrant emotions and behaviors are subserved by measurable physiological processes [24]; studying them could provide novel mechanistic insights into the psychopathology of irritability in youth. By examining psychophysiology during exposure sessions, we can probe the dynamic processes underlying adaptation to challenge in real-time [55], and how they vary within and between individuals over the course of treatment.

## Methods

The proposed study investigates peripheral psychophysiological changes within and between youth undergoing exposure-based CBT for clinically impairing irritability. The experimental protocols described herein have been approved by the Institutional Review Board at the National Institutes of Health (NIH Clinical Study Protocols 15-M-0182 (Clinicaltrials.gov identifier: NCT02531893)).

### Participants

Participants will be recruited locally (Maryland, District of Columbia, Virginia; USA) via mailings to selected physicians, announcements in newsletters, contacts with support groups and approved websites, advertisements and animated videos on social media, flyers and ads in public settings, and targeted mailings to households with children. We will follow n=40 youth (all genders; ages 8-17) over 6 in-person exposure sessions as part of a multiple-baseline trial of 12 weeks of exposure-based CBT for youth with clinically impairing irritability [73]. All participants and their legal guardians will provide informed assent (youth)/consent (parents/guardians). Eligible youth will be English-speaking, willing and able to provide informed assent with parental/caregiving informed consent, and present with at least one of two core symptoms of DMDD: abnormal mood or increased reactivity to negative emotional stimuli, with severe impairment in one domain (home, school, peers) and at least mild impairment in another, or moderate impairment in at least two domains [73]. Given the transdiagnostic nature of irritability, participants may have a primary diagnosis or comorbid diagnoses of DMDD, oppositional defiant disorder (ODD), ADHD, anxiety disorder(s), or other related disorders. Symptoms and diagnoses for inclusion and exclusion criteria will be established using the Kiddie-Schedule for Affective Disorders and Schizophrenia Present and Lifetime Version [45] with the additional DMDD supplement. Exclusion criteria include: (1) meeting current or past criteria for bipolar I/II disorder or any psychotic disorder; (2) persistent depressive disorder or current major depressive episode, PTSD, autism spectrum disorders (assessed using the Development and Well-Being Assessment [27], the Social Responsiveness Scale [18], the Social Communication Questionnaire [84], and the Children’s Communication Checklist-Second Edition [8, 76]; (3) IQ less than 70 as assessed by the Wechsler Abbreviated Scale of Intelligence (WASI; Vocabulary and Matrix Reasoning Scales) [106]; (4) a significant general medical or neurological condition; (5) meeting criteria for alcohol or substance use disorder within the last three months; and (6) conditions or life situations that would interfere with the participant’s ability to participate in treatment. Participants with past major depressive disorder will not be excluded. Given the parameters of the overarching clinical trial, participants taking medications (e.g., serotonin-selective reuptake inhibitors, anticholinergics, amphetamines, allergy medications, etc.) that could impact cardiovascular and electrodermal activity will not be excluded. Rather, medication use will be considered as a candidate covariate in analyses. Enrolled participants’ medications (if any) will be written by a psychiatrist at the National Institute of Mental Health working with the study team, and no changes in medication will occur during treatment unless there is an acute clinical need. Eligible consented participants will be randomized to a staggered start schedule as part of the multiple-baseline design, with either a two-, four-, or six-week baseline period. All study visits will occur at a single site, at the National Institute of Mental Health in Bethesda, MD.

### Study design

The data for the present study come from a randomized multiple baseline trial of exposure-based CBT. Each participant is randomized to 2, 4, or 6 weeks of baseline observation prior to treatment beginning. This design controls for the effects of time and regression towards the mean. Randomization is performed in blocks of 10 participants with a 1:1 within-block ratio. The assignment sequence was created via a computer-based random number generator. Clinicians conducting assessments (see below) are blinded to the trial phase from commencement of the baseline period, and the blind will not be broken for the entire cohort until the completion of the trial. The exposure-based CBT protocol will consist of twelve weekly sessions. Sessions are administered by two expert clinical psychologists. In-person exposure sessions (~30-45 minutes) will occur at weeks 5-10 in the protocol. Peripheral psychophysiological data will be collected during these six in-person exposure sessions. The main outcomes we plan to assess are changes in HR, HRV, and EDA over the course of exposure sessions and relations between changes in psychophysiological data and clinical symptoms. Clinical symptoms will be assessed weekly by clinician raters, including at mid-treatment, post-treatment, and 3- and 6-months post-treatment follow-up. Group supervision is conducted by the treatment developer and clinicians rate themselves on adherence after each session in order to improve adherence and minimize drift. Patients who request to discontinue or whose condition worsens (as determined by the clinical team and PI) may meet criteria for discontinuation. All participants are informed of their ability to discontinue at any time for any reason without the loss of benefits to which they are other entitled. For more extensive details regarding the intervention, please see Naim et al., 2021 and [69].

### Materials and measures

#### Questionnaires and clinical assessments

Assessments are performed by trained and liscensed masters- or doctoral-level clinicians who are blinded to the treatment. Clinical outcome measures will include the Clinician Affective Reactivity Index (CL-ARI [33];) to measure changes in irritability, and the Clinical Global Impressions-Severity (CGI-S) and CGI-Improvement (CGI-I) scales to measure overall illness severity and improvement [14]. Child- and parent-reported irritability will also be assessed using the child and parent-report versions of the ARI. The CGI-S will be the primary indicator of illness severity in analyses. Secondary analyses may also explore patterns of change and predictors of improvement based on the ARI, which includes not only the clinician but also the parent and child reports.

The CGI-S assesses the severity of psychopathology (here, irritability) on a 1-7 Likert scale where 1 = normal, not at all ill, and 7 = among the most extremely ill patients [14]. The CGI-I assesses change from the start of treatment on the same 7-point Likert scale, where 1 = very much improved and 7 = very much worse.

The CL-ARI is a reliable and valid measure comprising three subscales for temper outbursts, irritable mood between outbursts, and impairment [33]. Items are scored on Likert scales where higher scores indicate more severe, longer duration, and greater frequency of outbursts, mood, and impairment. Similarly, parent- and child-report versions of the ARI (six symptom items and one impairment item) show excellent internal consistency across samples (A [89, 91].).

#### Psychophysiology

The validity of psychophysiological measures is enhanced when they are measured during psychotherapeutic sessions that model the context in which individuals manifest their most severe symptoms through exposures [98]. We will record in-vivo psychophysiology using the Empatica EmbracePlus, a research-grade wearable biosensor that continuously collects and stores electrodermal activity (EDA; galvanic skin response) and photoplethysmography (PPG; HR/HRV). PPG is an optical technique used to measure blood volume changes in the microvascular bed of tissue [2]. The Empatica EmbracePlus uses two sets of photodiodes, each containing green and red LED operation wavelengths, to measure the light differences between oxygenated and non-oxygenated peaks. The Empatica EmbracePlus is an upgraded version of the Empatica E4, which has been validated against gold-standard tools for measuring HR and HRV. It is also valid and reliable for measuring HR and HRV in youth from clinical and non-clinical populations [85]. More broadly, previous studies have provided support for the use of wearable biosensors, like the Empatica devices as well as the Apple Watch and Fitbit, to provide valid and reliable indices of HR and HRV [39, 56, 66, 102]. The Empatica devices are supported by empirical research not only in adults but also in youth and provide users access to raw data from which HRV metrics can be derived in an event-related fashion. Empatica is unique from other devices in that participant data is fully anonymized and data loss is minimized due to onboard memory, onboard data processing, and continuous data streaming. In addition to EDA and PPG, Empatica devices measure skin surface temperature using thermopile and physical activity via three-axis accelerometry.

All in-person exposure sessions will occur in the same clinical space with a fixed ambient temperature. Participants will be seated during recordings, except for exposures that require them to move about the space. Participants will be asked not to consume caffeine before their visits and will be offered water to consume before the session begins. Participants will also be asked to wash their hands to ensure a clean surface for recording from the skin.

### Sample size and power analysis

The sample size for the overarching multiple baseline trial of exposure-based CBT was previously determined by [73] using pilot treatment data [47] and established a priori as n = 40. Based on preliminary data from n = 7 participants, n = 40 achieves power = 52.7% at alpha=0.05 for hypothesis 1, power = 29.7% at alpha = 0.05 for hypothesis 2, and power = 31.0% at alpha = 0.05 for hypothesis 4. However, given that these power analyses were based on a small pilot sample, they may be unreliable. A similarly designed study of changes in HR and HRV over the course of CBT identified significant effects in a sample of n = 43 [25]. Data from the present study will be used to inform the design of higher-powered studies to further explore the mechanistic predictors and underpinnings of this novel treatment.

### Preprocessing

#### HR/HRV

Blood volume pulse (BVP) data collected by the EmbracePlus is continuously analyzed by proprietary algorithms to extract digital biomarkers, including inter-beat intervals for measuring of HRV. Inter-beat intervals (IBIs) reflect the time between successive R-waves (i.e., RR time intervals) from ECG readings; for PPG, IBI reflects the time interval between successive pulsations, i.e., the pulse wave. Both R-waves and pulse waves serve as proxy measures of arterial depolarization resulting from SA-node action potentials. Because P-wave signal-to-noise ratio is poor for ECG, R-waves will be used instead. For PPG, the pulse wave, which proceeds the QRS complex (reflecting the ventricular contraction of the heart) by a slight delay, will be used. Data is sampled at a rate of 64 Hz.

We will use Kubios HRV Scientific to preprocess IBI data output from Empatica (derived from the BVP signal). Kubios is considered the gold-standard software for HRV analysis in both research and professional settings [95]. For each participant, sex, age in years, height in centimeters, weight in kilograms, resting HR in beats per minute, and maximum heart rate (the maximum HR value from the participant’s HR file exported from Empatica) will be entered into Kubios. Automatic noise detection will not be performed, and no noise segments will be removed or edited in Kubios since Empatica already removes erroneous peaks due to motion artifacts with their algorithm when calculating IBI. Additionally, motion artifacts may be induced by the exposures themselves, which is the clinical condition of interest. Finally, one study found that the highest validity was achieved when automatic noise detection and artifact removal were not used [85]. To mitigate excessive noise and motion artifacts, we will apply a wristband over the Empatica device to reduce light interference and participant preoccupation. We will use Kubios’s automatic beat correction and will record the number and percentage of corrected beats to potentially control for in analyses. Additionally, we will calculate the area under the curve as an indicator of overall session motion (based on actigraphy data recorded by EmbracePlus) and examine whether motion significantly differs within and between participants, to be considered as a possible covariate in analyses. The following data points will be extracted from Kubios: mean and standard deviation of heart rate (in beats per minute), RR time intervals (in milliseconds), minimum and maximum heart rate (in beats per minute), SDNN (in milliseconds), and RMSSD (in milliseconds). There are several metrics for HRV that map onto different physiological features. The RMSSD is a time-domain measure of cardiovascular activity [107]. The standard deviation of the normal-to-normal beat interval (SDNN) is another time-domain measure that captures the overall adaptability and flexibility of the system [55]. We will focus on SDNN as the primary indicator of HRV for this study. RMSSD will also be considered as a secondary indicator. We will only explore components from the time-varying domain of the signal, given the shorter duration of our recordings (1 hour or less treatment sessions) and the method of recording (PPG, as opposed to ECG).

#### EDA

We will use LedaLab to preprocess EDA data output from Empatica. Low- and high-pass filters will be applied to ensure that all data is within acceptable physiological ranges (0.01 to 100 microsiemens) following an adapted quality assessment protocol for ambulatory EDA data before moving on to analysis [49]. Empatica EDA is sampled at a rate of 4Hz. We will derive summary statistics of SCL (the average level of arousal across the entire session), change in SCL (magnitude and slope), SCR (range and maximum amplitude from the session), and number of SCR per minute.

### Data analysis

Extensive details of data management procedures can be found in Naim et al., 2021 and [69]. All participant data is de-identified using a numeric code to ensure confidentiality before, during, and after the trial. Only approved staff at the study site (National Institute of Mental Health) will have access to data. Estimates for HR, HRV, and EDA will be calculated as the session average for each individual across all sessions. Given the tendency of physiological data to be non-normally distributed, we will log-transform HR, HRV, and EDA data as necessary [55]. Questionnaire data will also be screened for normality and univariate outliers.

Multilevel models will be fit. Sessions (time; level 1) will be nested within person (level 2). Psychophysiological variables (HR, HRV, and EDA) from session 1 will be included at both levels 1 and 2 to account for the effects of individual differences at baseline on the intercept and slope, allowing us to test whether individual differences are predictive of treatment response within this model. Linear, quadratic, and cubic fits will be compared to determine the best-fitting model of change over treatment. First, we will sequentially fit models with psychophysiological indicators (HR, HRV, or EDA) from each of the five in-person exposure sessions defined as the outcome variable to assess change over time. Second, we will model severity of irritability based on the CGI as the outcome variable, and psychophysiological variables will be grand mean centered and entered as predictors at level 2 to assess the relation between change in psychophysiological parameters over time and irritability at post-treatment, 3-month follow-up, and 6-month follow-up. Missing data will be assessed for systematic loss and will be handled using full information maximum likelihood, which can handle both missing data and unequal time between measurement intervals [38]. We will use the Bonferroni-Holm method to correct for multiple comparisons.

In addition to the dimensional models described above, wherein irritability and change in irritability are conceptualized continuously, we will also fit dichotomous treatment response models. Individuals will be classified as either high or low responders per the methodology described in [60]. High responders will be defined as those individuals who demonstrate a 50% or greater reduction in irritability based on the CGI at post-treatment assessment. Multilevel models will be fit as described above, with responder status as the outcome variable.

Should our primary hypothesis testing reveal changes in arousal (HR/EDA) and regulation (HRV) over the course of in-person exposure sessions, we will perform post hoc exploratory analyses to investigate the temporal relationship between psychophysiology and change in symptoms over the course of treatment using cross-lagged panel modeling. To capture weekly variation in symptoms, we will use the CGI rated at each exposure session. A random intercept cross-lagged panel model (RI-CLPM) will be specified in R using the lavaan package [82]. Four components will be specified: (1) a between component, consisting of random intercepts; (2) within-person fluctuations; (3) the lagged regressions between within-person components; and (4) covariances of the within and between components [68]. Within-person variables will be mean-centered.

## Discussion

This study protocol tests peripheral psychophysiological mechanisms that may underly exposure-based CBT for youth with clinically impairing irritability. The current protocol leverages state-of-the-art measures by collecting ambulatory psychophysiological data in the context of treatment via a medical-grade device. Psychophysiological data will be evaluated alongside intensive, weekly clinical ratings from patients, parents, and trained clinicians. Despite the strengths and innovative aspects of the current study, the multiple-baseline trial is not a randomized controlled trial; therefore, it does not allow for strong causal inferences.

In-session measurement of psychophysiology over the course of treatment could provide a more nuanced understanding of mechanisms underlying exposure-based CBT for clinically impairing irritability, as well as predictors of treatment outcomes [108].

## Data Availability

We plan to share datasets and scripts from the study described herein in a publically accessibly database; at this time data sharing is not applicable as no data is described herein. Study findings will be disseminated through publications in peer-reviewed journals, presentations at national and international conferences, presentations to local community partners (e.g., school districts, parent-teacher associations, community providers, and advocacy organizations), on the lab’s website, and through a quarterly newsletter distributed to lab member and study participants.

## Abbreviations

CBT: Cognitive behavioral therapy
HR: Heart rate
EDA: Electrodermal activity
HRV: Heart rate variability
BVP: Blood volume pulse
CGI: Clinical Global Impressions Scale
ARI: Affective Reactivity Index
NIMH: National Institute of Mental Health
ADHD: Attention-deficit/hyperactivity disorder
DMDD: Disruptive mood dysregulation disorder
DSM: Diagnostic and Statistical Manual
ANS: Autonomic nervous system
vmPFC: Ventromedial prefrontal cortex
SNS: Sympathetic nervous system
PE: Prolonged exposure
PTSD: Posttraumatic stress disorder
RMSSD: Root mean square of successive differences
BOLD: Blood oxygen level-dependent
SDNN: Standard deviation of the normal to normal beat interval
USA: United States of America
ODD: Oppositional Defiant Disorder
WASI: Wechsler Abbreviated Scale of Intelligence
CL-ARI: Clinician Affective Reactivity Index
CGI-S: Clinical Global Impressions-Severity
CGI-I: Clinical Global Impressions-Improvement
PPG: Photoplethysmography
IBI: Inter-beat interval
RR: Successive R waves
SA: Sinoatrial
ECG: Electrocardiogram
RI-CLPM: Random intercept cross-lagged panel model
